# Prognostic value of quantitative EEG in early hours of life for neonatal encephalopathy and neurodevelopmental outcomes

**DOI:** 10.1038/s41390-024-03255-8

**Published:** 2024-07-22

**Authors:** Srinivas Kota, Shu Kang, Yu-Lun Liu, Hanli Liu, Saeed Montazeri, Sampsa Vanhatalo, Lina F. Chalak

**Affiliations:** 1 Division of Neonatal-Perinatal Medicine, Department of Pediatrics, University of Texas Southwestern Medical Center, Dallas, TX USA; 2 Department of Bioengineering, University of Texas at Arlington, Arlington, TX USA; 3 Peter O’Donnell Jr. School of Public Health, University of Texas Southwestern Medical Center, Dallas, TX USA; 4 Department of Physiology, University of Helsinki, Helsinki, Finland

## Abstract

**Background:**

The ability to determine severity of encephalopathy is crucial for early neuroprotective therapies and for predicting neurodevelopmental outcome. The objective of this study was to assess a novel brain state of newborn (BSN) trend to distinguish newborns with presence of hypoxic ischemic encephalopathy (HIE) within hours after birth and predict neurodevelopmental outcomes at 2 years of age.

**Method:**

This is a prospective cohort study of newborns at 36 weeks’ gestation or later with and without HIE at birth. The Total Sanart Score (TSS) was calculated based on a modified Sarnat exam within 6 h of life. BSN was calculated from electroencephalogram (EEG) measurements initiated after birth. The primary outcome at 2 year of age was a diagnosis of death or disability using the Bayley Scales of Infant Development III.

**Results:**

BSN differentiated between normal and abnormal neurodevelopmental outcomes throughout the entire recording period from 6 h of life. Additionally, infants with lower BSN values had higher odds of neurodevelopmental impairment and HIE. BSN distinguished between normal (*n* = 86) and HIE (*n* = 46) and showed a significant correlation with the concomitant TSS.

**Conclusion:**

BSN is a sensitive real-time marker for monitoring dynamic progression of encephalopathy and predicting neurodevelopmental impairment.

**Impact:**

- This is a prospective cohort study to investigate the ability of brain state of newborn (BSN) trend to predict neurodevelopmental outcome within the first day of life and identify severity of encephalopathy.
- BSN predicts neurodevelopmental outcomes at 2 years of age and the severity of encephalopathy severity. It also correlates with the Total Sarnat Score from the modified Sarnat exam.
- BSN could serve as a promising bedside trend aiding in accurate assessment and identification of newborns who may benefit from additional neuroprotection therapies.

## Introduction

Hypoxic ischemic encephalopathy (HIE) is one of the leading causes of mortality and morbidity in newborns worldwide.^1^ HIE occurs when the fetal brain is deprived of oxygen and blood supply^2^ and the encephalopathy severity is classified as mild, moderate, or severe grades within 6 h after birth.^3–5^ While therapeutic hypothermia (TH)^6^ is standard clinical care for moderate and severe grades in developed countries, the management of mild HIE is controversial^7^ due to difficult clinical classification and progression of encephalopathy grade. Mild HIE accounts for 50% of total HIE.^8^ Newborns with mild HIE and worsening symptoms in the first day of life are often treated with TH using a late hypothermia protocol, but its effectiveness may be limited if it is initiated after 6 h of life.^9^

Electroencephalogram (EEG) monitoring is routinely used to detect seizures and predict outcomes in HIE.^10^ We and others have studied qualitative and quantitative metrics, such as background EEG activity, amplitude-integrated EEG (aEEG), spectral power, and connectivity, for prognosis.^11–16^ However, due to the lack of continuous interpretation, these biomarkers have not been available to be used for timely interventions.

Montazeri et al. proposed the brain state of newborn (BSN) as a novel monitoring visual trend, which uses deep learning-based EEG classifier to quantify EEG background in infants recovering from birth asphyxia.^17^ This method achieved 92% accuracy and was validated with an external EEG dataset.

This study aims to evaluate the association between automatically computed BSN trends within the first 6–9 h of life in newborns with HIE and their neurodevelopmental impairment (death or disability) at the age of 2 years. Additionally, the study examines the association between BSN trends and the severity of encephalopathy, as well as the relationship with the total Sarnat score (TSS) obtained from the modified Sarnat exam. Our hypothesis posits that lower BSN values may indicate potential neurodevelopmental impairment, while higher TSS values may be associated with lower BSN values. Furthermore, we hypothesize that BSN can accurately and quickly differentiate newborns with encephalopathy based on its severity on the first day of life, which could help identify those who would benefit from neuroprotective therapies.

## Methods

### Study participants

#### HIE cohort

Term newborns (≥36 weeks’ gestation) at Parkland Hospital (Dallas, TX) who met the following criteria were recruited for this prospective cohort study between 2017 and 2019: (1) a history of an acute perinatal event (e.g., placental abruption, cord prolapse, decreased fetal heart rate), (2) umbilical cord arterial pH or arterial blood gas pH of ≤7.0 or base deficit ≥15 mmol/L at <1 h postnatal age, and (3) signs of encephalopathy. Newborns were excluded from the study if they had any genetic or congenital condition, birthweight < 1800 g, and/or head circumference <30 cm, as these factors can interfere with the primary outcome. The study was approved by the Institutional Review Board at University of Texas Southwestern Medical Center, and written informed consent was obtained from a parent of each newborn prior to enrollment.

The newborns were evaluated within 6 h after birth using a modified Sarnat exam by trained clinicians to determine the severity of encephalopathy that included (1) level of consciousness, (2) spontaneous activity, (3) posture, (4) tone, (5) primitive reflexes (suck, moro), and (6) autonomic system (pupils, heart rate, respirations), with scores of normal (0), mild (1), moderate (2), or severe (3). The TSS was determined by adding the scores for each of the six categories, which ranges from 0 to 18, where 0 represents normal in all six categories, and 18 represents severe encephalopathy in all six categories.^5^ The clinical grade of encephalopathy was determined by the number of Sarnat abnormalities, with classifications ranging from mild to moderate or severe. In cases where equal numbers of abnormalities were observed, the grade was determined by the degree of reduced level of consciousness. The TSS scores were obtained from electronics medical records.

Whole-body TH was initiated within 6 h after birth for newborns with moderate and severe encephalopathy, following the National Institute of Child Health and Human Development (NICHD) protocol.^6^ A servo-controlled blanket (Blanketrol II, Cincinnati Sub-Zero Products LLC, OH) was used to maintain a core body temperature of 33.5 °C for 72 h, followed by rewarming at a rate of 0.5 °C per 1–2 h for the next 6 h. Newborns with mild encephalopathy received normothermia as per the standard of care. TH was initiated in accordance with the NICHD late hypothermia protocol if they progressed to more severe encephalopathy or experienced seizures within the first day of life.^9^

Neurodevelopmental impairment at age of 2 years was death or disability defined by a cognition, language, or motor score <85 on the Bayley Scales of Infant Toddler Development, third edition (BSID-III),^5,18,19^ which was performed by certified professionals in the follow up clinic at 18–24 months of age.

Continuous EEG acquisition was initiated at a sampling rate of 256 Hz as soon as newborns were admitted, following parental consent. EEG (Nihon Kohden America Inc., Irvine, CA) data were acquired from eight scalp electrodes (Fz, C3, Cz, C4, P3, P4, O1, O2) that were referenced to the Pz, placed according to the modified 10-20 montage for newborns.^20^ The Component Neuromonitoring System (CNS) Monitor (Moberg Research, Inc., Ambler, PA) was used as the bedside interface for EEG (Nihon Kohden America Inc., Irvine) and other physiological signals, including Near-Infrared Spectroscopy (NIRS) from the INVOS™ 4100–5100 oximeter (Somanetics, Troy, MI), the Blanketrol cooling device, and the Philips IntelliVue MP70 for electrocardiography, mean arterial pressure, and peripheral capillary oxygen saturation (SpO_2_). The EEG data were obtained from CNS monitor and processed offline using MATLAB (MathWorks Inc., Natick, MA).

Central and parietal electrodes were chosen for analysis because they reflect watershed injury patterns on MRI in HIE.^21–23^ Inter- (C3-C4, P3-P4) and intra- (C3-P3, C4-P4) hemispheric bipolar EEG data were obtained by taking a difference between pair of electrodes. The bipolar EEG data were high pass filtered at 0.3 Hz using a Butterworth filter of order 4.

#### Control cohort

The control cohort was collected retrospectively from the clinical archives in the Department of Clinical Neurophysiology, New Children’s Hospital, University of Helsinki. The EEG recordings used for the present purpose were part of a larger re-assessment of EEG recordings acquired and reviewed between 2011 and 2016. Here, we included all infants that were born at term age and the EEG was recorded for clinical indications (e.g., Exclusion of seizure activity), but the EEG report was fully normal and there were no neurological consequences found in the comprehensive review of the patient reports. All EEG data was recorded using a NicoletOne system (Cardinal Healthcare/Natus) at sampling rate of 256 Hz from four need electrodes (F3, F4, P3, and P4). All the bipolar signals (F3-P3, F4-P4, F3-F4, and P3-P4) were obtained for further analysis. This data collection was approved by the Institutional Research Review Board at Helsinki and Uusimaa Hospital district approved the study (HUS/244/2021) including waiver of consent due to the retrospective collection of data acquired as part of standard of care.

### Brain state of newborn (BSN)

An automated cloud service tool (https://babacloud.fi/) was employed to calculate BSN from aEEG from bipolar combinations, and identify seizures and artifacts for every 2 s segments of data.^17^ The cloud service is fully automated and it needs no prior experience from the user, apart from uploading the EEG file, followed by downloading the analysis results for each EEG file. The analysis algorithm has been previously explained in full detail,^17^ including its rationale, technical design, training, and external validations. Montazeri et al.^17^ collected visually classified EEGs from newborns recovering from birth asphyxia or stroke.^24,25^ Unsupervised learning methods were used to explore latent EEG characteristics using a training dataset of 2561 h of multi-channel EEG recordings from 39 term newborns from previously published clinical cohorts.^24,25^ These insights guided the supervised training of a deep learning-based classifier that achieved an accuracy comparable to human inter-rater agreement. The BSN was calculated by combining the novel EEG background classifier and sleep state trend. The algorithm’s performance was validated using an external dataset^26^ consisting of 105 h of multi-channel EEG recordings from 31 newborns with HIE. Residual deep neural networks^27^ identified artifacts like device interference, electromyograph (EMG), movement, electrode pops, non-cortical rhythms like electrocardiogram (ECG), high amplitudes, and zeros. Hybrid neonatal EEG seizure detection algorithms were used to calculate seizure probability.^28^ Seizure detection probability greater than 0.5 considered as presence of seizure.

All raw analysis outputs from Babacloud are given as numerical vectors for each 2 s sample of the given EEG recording. All further analysis was done offline, including compression by averaging over 3-h epochs, and other statistical analyses. To maximize real-world utility and validity of the pipeline, the full EEG data was always analyzed, and the signal quality automatically assessed using the integrated artifact detector/classifier algorithm. This approach aimed to remove subjective components and other potential biases in the data selection. BSN is a continuous score that ranges from 0 (inactive EEG) to 100 (fully active), allowing a straightforward comparison to other clinical information, as well as avoiding the inherent ambiguity related to classification with discrete EEG scores.^17^ Artifact rejection was performed on every 1-min nonoverlapping segment of the BSN if they contained more than 30 s of artifacts or more than 6 s of seizure detections. Additionally, if at least two of the four electrodes in a 1-min segment were labeled as artifacts or seizures, the BSN value for the entire 1-min segment were removed from subsequent analysis. The mean BSN value was calculated from artifact-free segments within a 3-h segment for statistical analysis.

### Statistical analysis

Demographic and clinical characteristics of newborns, stratified by non-mild HIE (including mild-moderate, moderate, and severe) and mild HIE, were summarized with descriptive statistics, where continuous variables were presented as means with standard deviations or medians with interquartile ranges (IQRs), and categorical variables were presented with counts with percentages. The non-mild and mild HIE groups were compared using Student’s *t* test or Wilcoxon rank-sum test for continuous variables, while ${{{{{\rm{\chi }}}}}}^{2}$ test or Fisher’s exact test for comparisons among categorical variables. Univariate linear regression analysis was performed to determine the association between BSN and TSS. The assumptions for linear regression model, including normality of residuals and homoscedasticity, were evaluated using Shapiro-Wilk test and Breusch-Pagan test. Univariate logistic regression models were used to assess the relationships of BSN on Bayley-III neurodevelopmental outcomes, including 2-year Bayley, cognitive, language, and motor outcomes. To evaluate the prediction ability of BSN on Bayley-III neurodevelopmental outcomes, we conducted the receiver operating characteristic (ROC) curve, with the area under the ROC curve (AUC). The optimal cut-off values of BSN were obtained with the Youden method to distinguish between normal and abnormal neurodevelopmental outcomes in infants. Results were reported as odds ratios (ORs) with 95% confidence intervals in logistic regression models, and regression coefficients with 95% confidence intervals in linear regression models. A 2-tailed p value less than 0.05 was considered the threshold for statistical significance. No adjustments were made for multiple comparisons, thus our findings for secondary outcomes (including the severity of encephalopathy and TSS) and subsequent analyses (such as exploring different post-birth time windows both prior to and beyond 6–9 h) should be interpreted as exploratory. All statistical analyses were performed using R version 4.2.2.

## Results

A total of 46 newborns with symptoms ranging from mild to severe HIE, as determined by the modified Sarnat exam within the first 6 h after birth, were recruited for this study and compared with 86 reference control newborns. Among HIE cohort, 26 newborns were initially classified as mild in the first 6 h of life. However, seven of those newborns later developed seizures or displayed worsening symptoms of encephalopathy, leading to their reclassification as mild-moderate. Maternal and neonatal characteristics of the HIE cohort are provided in Table 1. The non-mild (mild-moderate, moderate, and severe) and mild group included 27 and 19 newborns respectively. Two newborns with severe grade died in the first week of life following redirection of care.

**Table 1:** Maternal and neonatal characteristics of the HIE cohort

Characteristics	Overall	Encephalopathy Grade		
		Mild	Non mild Cooled	*P* value
Total *N*	46	19	27	----
Male: *N* (%)	29 (63)	14 (74)	15 (56)	0.345
Gestational Age (weeks), median [IQR]	39 [38, 40]	39 [38, 40]	39 [38, 40]	0.691
Birth Weight (kg), mean (SD)	3.3 (0.7)	3.4 (0.6)	3.3 (0.8)	0.523
Apgar 1 min, median [IQR]*	2 [1, 4]	3 [2, 5]	1 [1, 2]	0.004
Apgar 5 min, median [IQR]*	6 [4, 7]	7 [6, 8]	4 [2, 6]	0.001
Umbilical Cord Gas pH, mean (SD)	7 (0.1)	7(0.1)	7(0.1)	0.656
Base Deficit, mean (SD)	17.0 (6.1)	17.4 (3.6)	17.0 (7.4)	0.730
Abnormal MRI (Global): *N* (%)	12 (26)	4 (21)	8 (30)	0.735
Maternal Race/Ethnicity: *N* (%)				
*Caucasian non-Hispanic*	2 (4)	1 (5)	1 (4)	>0.999
*Black non-Hispanic*	10 (22)	4 (21)	6 (22)	
*Hispanic*	31 (67)	13 (68)	18 (67)	
*Other non-Hispanic*	3 (7)	1 (5)	2 (7)	
Delivery Mode: *N* (%)				
*C/S*	29 (63)	12 (63)	17 (63)	>0.999
*Vaginal*	17 (37)	7 (37)	10 (37)	
Maternal Risk Factors: *N* (%)				
*Hypertension*	10 (22)	4 (21)	6 (22)	>0.999
*Diabetes*	5 (11)	4 (21)	1 (4)	0.144
*Pre-eclampsia*	13 (28)	4 (21)	9 (33)	0.510
Labor Complications: *N* (%)				
*Meconium*	12 (26)	2 (11)	10 (37)	0.086
*Umbilical Cord Prolapse*	1 (2)	0	1 (4)	>0.999
*Placental Abruption*	4 (9)	1 (5)	3 (11)	0.632
*Uterine Rupture*	4 (9)	2 (11)	2 (7)	>0.999
*Maternal Chorioamnionitis*	13 (28)	6 (32)	7 (26)	0.931
*Placental Chorioamnionitis*	24 (52)	10 (53)	14 (52)	>0.999
Disposition:				
DOL at discharge, median [IQR]*	9 [6, 17]	6 [5, 7]	14 [9, 20]	<0.001
Death prior to discharge, *N* (%)	2 (4)	0	2 (7)	0.504
Neurodevelopmental Outcome:				
Total N	38	13	25	----
Composite, death or disability *N* (%)	33 (87)	11 (85)	22 (88)	>0.999
BSID Score: median [IQR]				
Cognition	85 [75, 90]	83 [75, 95]	85 [80, 90]	0.874
Language	71 [61, 79]	73 [59, 80]	68 [62, 78]	0.862
Motor	94 [88, 100]	94 [78, 100]	94 [91, 102]	0.422

When the normality assumption was violated, median and interquartile range (IQR) were reported, and the Wilcoxon rank sum test was used to assess statistical significance.

*SD* standard deviation, *IQR* interquartile range, *DOL* days of life.

*Indicates statistical significance (*p* < 0.05).

The severity of encephalopathy was associated with lower Apgar scores and longer hospital stays (Table 1). There was no statistically significant difference in BSID-III scores between the mild and non-mild groups. An abnormal score in cognition, language, or motor skills was defined as a BSID-III score <85. For 2-year neurodevelopmental outcome, any of the three BSID-III scores <85 or death was considered abnormal. Normal newborns (conceptual age CA 40 [39, 42]) with no neurological abnormalities were included as a benchmark for the study cohort (CA 39 [38, 40]) and their neurodevelopmental outcome was recorded normal.

**Fig. 1:**
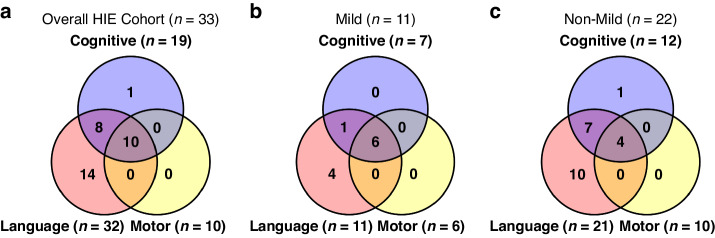
The Venn diagram illustrates number of infants with cognitive, language, and motor abnormalities. Overlapping regions represent infants with abnormalities in multiple categories. **a** Overall HIE Cohort: Ten out of 33 infants either died or had abnormal Bayley scores in all three categories. Eight had cognitive and language abnormalities, fourteen had language abnormalities, and one had cognitive abnormality. No infants had isolated motor impairment. **b** Mild HIE Cohort: Six out of 11 infants had abnormal Bayley scores in all three categories, one had cognitive and language impairments, and four had language impairment only. **c** Non-Mild Cohort: Four out of 22 infants either died or had impairment in all three categories. Seven had cognitive and language impairments, one had cognitive impairment alone, and ten had language impairment alone.

Figure 1 shows that there were some infants with more than one abnormality at 2 years of age. Among the 46 infants in the overall HIE cohort, 8 (17%) were lost to 2-year follow-up (6 mild, 1 mild-moderate, 1 moderate). Of the remaining 38, only 5 (13%) had normal neurodevelopmental outcome, while 33 (87%) had abnormal outcome. Among infants with abnormal outcomes as shown in Fig. 1a, 10 (30%) had impairments in all three key areas (cognition, language, and motor function), 8 (24%) showed impairments in both cognition and language, 1 (3%) had isolated cognitive impairment, and 14 (42%) had language impairment only.

Among 26 infants initially categorized as mild, 7 transitioned to the mild-moderate group within the first day of life. Among the remaining 19, six (32%) were lost to follow-up. Out of the 13 remaining infants in the mild group, two (15%) had normal neurodevelopmental outcome, while 11 (85%) had abnormal outcome. Within the mild HIE group as shown in Fig. 1b, six infants (55%) exhibited abnormalities across all three domains (cognition, language, and motor). One (9%) had abnormalities in both cognition and language, and four (36%) had abnormalities in language only.

Out of 27 infants with non-mild HIE, two (7%) were lost to follow-up. Among the 25 remaining infants, only three (12%) had normal neurodevelopmental outcomes, while 22 (88%) had abnormal outcomes. As shown in Fig. 1c, of the 22 infants with abnormal outcomes, four (18%) had significant abnormalities in all three areas (cognition, language, and motor function) or died. Seven (32%) showed abnormalities in both cognition and language, while one (5%) had isolated cognitive abnormality and ten (45%) had language abnormality alone.

There is no statistically significant difference in the number of infants between the mild and non-mild groups. To enhance the predictive power of our model for identifying infants at high risk of neurodevelopmental impairment, we combined data from the control cohort (*n* = 86) with normal outcomes from the HIE group (*n* = 5).

TH was initiated at a mean ± SD of 5.160 ± 3.948 h of life (HOL) in the HIE cohort. Specifically, for newborns with moderate or severe HIE, the initiation time was 4.343 ± 1.04 HOL, while in the mild-moderate (late hypothermia) group, it was 7.492 ± 7.451 HOL.

The EEG recording for the entire HIE cohort initiated at a mean ± SD of 13.241 ± 6.598 HOL and concluded at 72.230 ± 35.512 HOL. In the mild HIE subgroup, the initiation of EEG recording occurred at 12.415 ± 6.247 HOL, with recording concluding at 40.913 ± 11.530 HOL. Conversely, for the non-mild HIE (TH) subgroup, EEG recording started at 13.823 ± 6.890 HOL and concluded at 94.268 ± 29.489 HOL.

The EEG recording duration for the HIE cohort was 58.989 ± 34.742 h. Specifically, for the mild HIE group, duration was 28.499 ± 12.468 h, while for the non-mild HIE (TH) group, it was 80.445 ± 28.706 h. Due to the limited number of newborns with HIE before 6 h of life and beyond 60 h after birth, the analysis was confined to the period between 6 and 60 h after birth. A total of 4.068 ± 5.833% of 1-min segments were excluded from the data analysis due to the presence of artifacts or seizures.

**Fig. 2:**
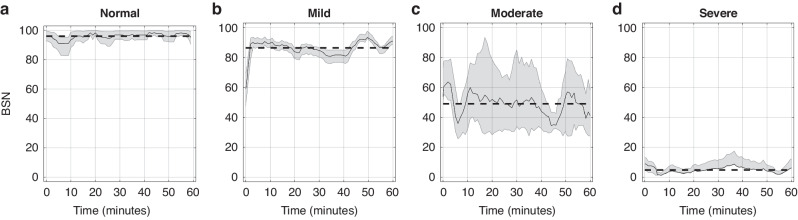
BSN trends in control and HIE cohorts. BSN trends for normal (**a**) and newborns with encephalopathy grades mild (**b**), moderate (**c**), and severe (**d**), during the first hour of recording. The shading in the figure represents the classifier’s confidence in the corresponding BSN values. The dashed line indicates the mean trend value. There is a clear difference in mean trend values between newborns with different encephalopathy grades.

**Fig. 3:**
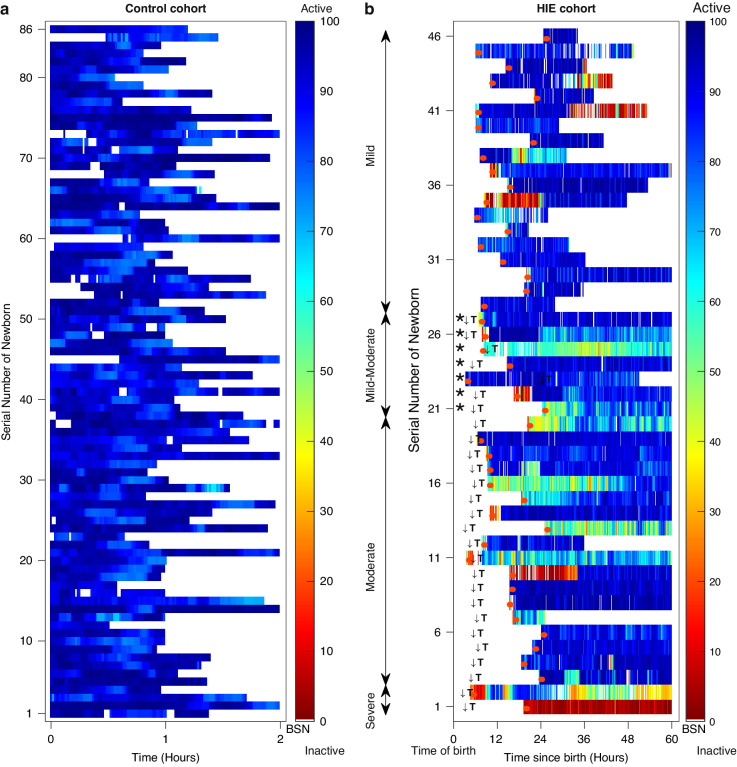
BSN values in control and HIE cohorts: BSN values were calculated for every 2-s EEG segment using inter- and intra-hemisphere bipolar electrodes. **a** The BSN values for each newborn in the control cohort are shown over 2 h in each row. **b** The BSN values for each newborn in the HIE cohort, with severity ranging from severe to mild (from bottom row to top row), are shown over the first 60 h of life in each row. Newborns with an asterisk (*) were initially classified as mild but later progressed to moderate. The color bar shows arbitrary units, ranging from 0 (inactive brain activity) to 100 (normal brain activity). White spaces indicate missing data, which may be caused by artifacts, seizures, or unavailability of data. Initiation of TH is indicated with ↓T and beginning of EEG acquisition is indicated with an orange color.

The BSN trajectories for a single newborn across the first hour of recording, categorized by encephalopathy grades (A-normal, B-mild, C-moderate, D-severe) are shown in Fig. 2. Distinct mean BSN trends were observed between newborns with varying encephalopathy severities. Figure 3a, b illustrates clear visual distinction between normal newborns BSN values compared to the HIE cohort.

**Fig. 4:**
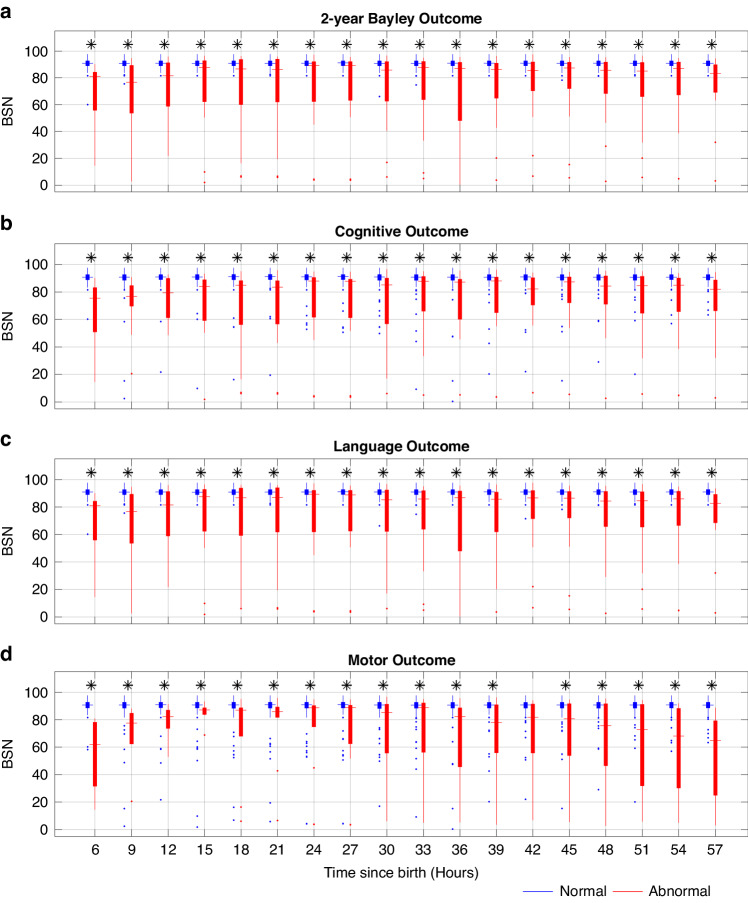
Distribution of mean BSN values. Distribution of mean BSN values over 3-h windows from 6 to 60 h hour since birth for normal and abnormal 2-year Bayley (**a**), cognitive (**b**), language (**c**), and motor (**d**) outcomes. Abnormal outcome was defined as death or 2-year Bayley scores < 85 in any of cognitive, language, or motor domains. An asterisk on the top of each figure indicates that the BSN values for infants with normal outcome are significantly greater than those for infants with abnormal outcomes, as determined by the Wilcoxon rank-sum test.

**Fig. 5:**
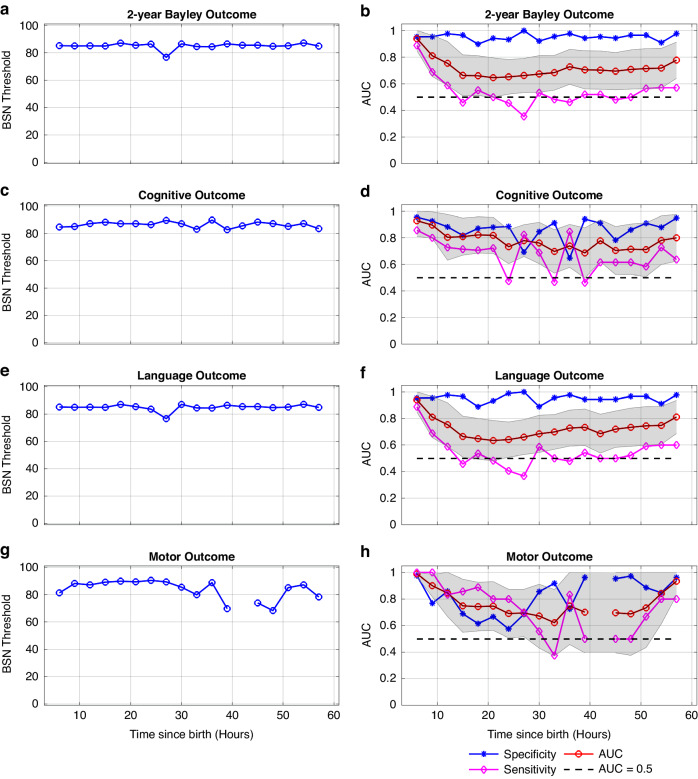
Receiver operating characteristic (ROC) analysis was performed to identity the optimal BSN threshold for distinguishing normal from abnormal 2-year Bayley, cognitive, language, and motor outcome at different time points since birth (6–60 h). The left panel (**a**, **c**, **e** and **g**) shows that a BSN value over 85 is the most effective discrimination between the two groups. The right panel (**b**, **d**, **f** and **h**) shows that the area under the curve (AUC) for all outcomes and time points except motor at 42–45 h is greater than 0.5. The shaded region indicates the 95% CI of the AUC.

Distribution of BSN values for every 3-h time window for normal and abnormal outcomes are shown the Fig. 4 for 6–60 h since birth. The results of ROC curves for 2-year Bayley, cognitive, language, and motor outcomes at the age of 2 years are presented in Fig. 5. In Fig. 5, it is evident that a BSN value of 85 effectively differentiated abnormal 2-year Bayley outcome from normal, achieving sensitivity of 0.889 and specificity of 0.954 at 6–9 h since birth. The corresponding AUC value was 0.940 (95% CI, 0.850–1.0). Comparable findings were observed for 2-year Bayley, cognitive, language, and motor outcomes for 6–60 h after birth as shown in Fig. 5.

**Fig. 6:**
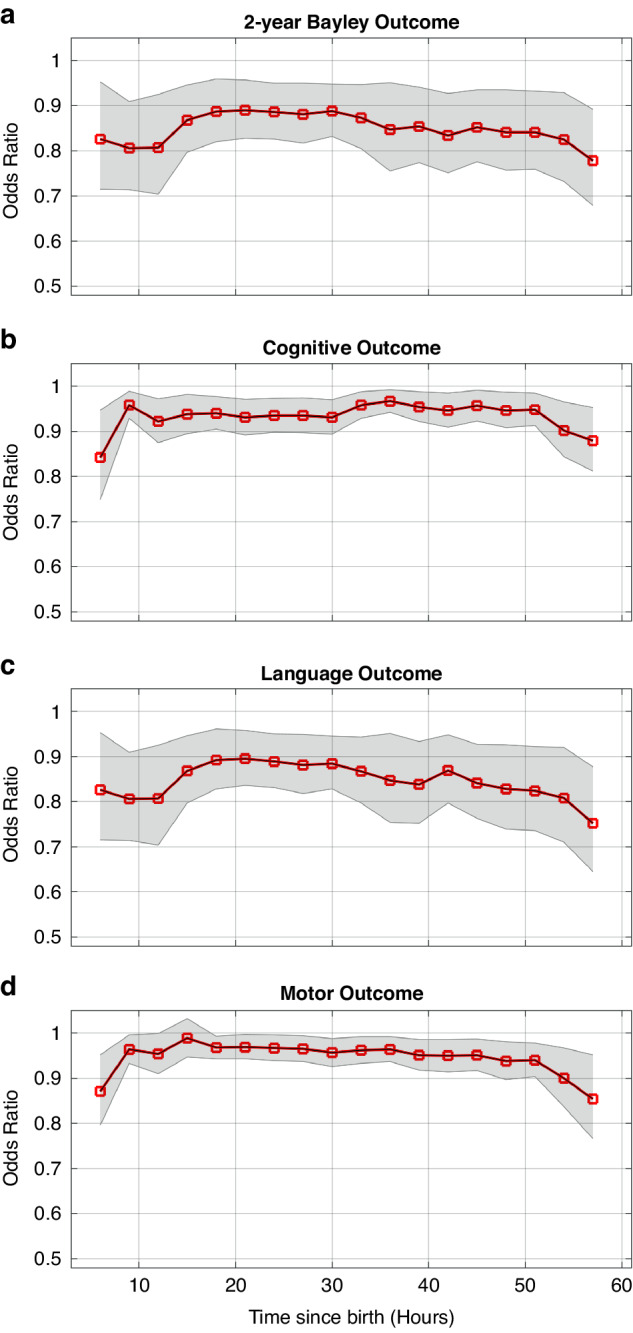
Univariate logistic regression analysis: Odds ratios (ORs) for global, cognitive, language, and motor outcomes at different time points since birth (6–60 h) are shown in 3-h windows. Shaded areas indicate 95% confidence intervals (CIs) for ORs. Lower BSN values were significantly associated with higher odds of abnormal 2-year Bayley (**a**), cognitive (**b**), language (**c**), and motor (**d**) outcomes.

In addition, univariate logistic models reveal significant relationships between BSN and 2-year Bayley, cognitive, language, and motor outcomes. Lower BSN values are associated with higher odds of abnormal 2-year Bayley (OR, 0.826; 95% CI, 0.715–0.953; *p* < 0.001), cognitive (OR, 0.842; 95% CI, 0.749–0.947; *p* < 0.001), language (OR, 0.826; 95% CI, 0.715–0.953; *p* < 0.001), and motor (OR, 0.871; 95% CI, 0.796–0.952; *p* < 0.001) outcomes at 6–9 h after birth. Similar trend of ORs can be observed in Fig. 6 for 6–60 h after birth.

The BSN value measured between 6 and 9 h after birth was used to classify HIE vs. normal. The area under the curve (AUC) for the BSN value was 0.885 (95% CI, 0.746–1.000), a sensitivity of 0.769 and a specificity of 0.965. A BSN value of 85 was found to effectively differentiate HIE from normal. A BSN cutoff of 68 was able to distinguish between non-mild and mild HIE, yielding an AUC of 0.738 (95% CI, 0.397–1.000), with a sensitivity of 0.667 and a specificity of 1. Univariate logistic regression analysis showed that a lower score of BSN was associated with increased odds for the occurrence of HIE (OR, 0.669; 95% CI, 0.533–0.839; *p* < 0.001). Subgroup analysis of mild vs. non-mild HIE did not show statistical significance in the univariate logistic regression analysis (OR 0.903; 95% CI, 0.799–1.020; *p* = 0.101).

In the univariate linear regression model that assessed the effect of BSN on TSS, BSN was negatively correlated with TSS, and the model explained 17% of the variation of TSS (i.e., ${{{{{\rm{R}}}}}}^{2}$ of 0.17). It was found that for every 1-unit increase in BSN, TSS was decreased by 0.071 points (95% CI, −0.118 to −0.024; *p* = 0.005). The Shapiro-Wilk test (*p* = 0.122) and the Breusch-Pagan test (*p* = 0.213) suggested the normality of the residual and homoscedasticity, respectively.

## Discussion

This study highlights BSN as a promising bedside marker to determine brain health, predict neurodevelopmental outcome and severity of encephalopathy using the first hours of EEG data since birth. This is especially crucial when the neurological exam may not accurately determine the severity of encephalopathy within the first 6 h after birth, particularly for identifying dynamic mild encephalopathy cases that would benefit from neuroprotection therapies. Furthermore, findings show the BSN has promising potential to quantify evolving encephalopathy in the first day of life.

BSID-III scores did not show a statistically significant difference between the mild and non-mild HIE groups. Similar findings were reported by Finder et al.^29^ where the BSID cognitive scores in the mild group untreated with TH and the moderate group that received TH were not statistically different. Lower BSN values are significantly associated with higher odds of abnormal neurodevelopmental outcome. ROC and logistical regression analysis were based on average BSN over 3 h, but little or no difference was observed when BSN values were overaged over shorter time intervals (every 1–2 h) from 6 to 60 h after birth (Supplementary Figs. S1–S5). To account for the difference in conceptual age between the control and HIE cohorts, logistical regression analysis was adjusted for conceptual age. However, little to no difference in ORs was observed after adjusting conceptual age (Supplementary Figs. S6, S7).

Heterogeneity across various encephalopathy grading systems like Sarnat, modified Sarnat exam, NICHD, and SIBEN, highlight the need of a standardized automated EEG quantitative system.^30^ For instance, newborns, with mild encephalopathy a TSS ranging from 1 to 10 is possible, while moderate ranges from 6 to 14, and severe from 9 to 18. Chalak et al. reported in PRIME study that a TSS value of $\ge$5 within first 6 h of life predicted neurodevelopmental impairment and encephalopathy burden.^5^ The correlation between BSN and TSS is similar to the correlation between EEG delta power and TSS (C3-C4 regression coefficient −0.010, *R*^2^ = 0.17, *p* = 0.006).^12^ This suggests that BSN trend and delta power may be two different ways to assess severity of encephalopathy when trained and certified professionals are unavailable to conduct a neurological exam within the first 6 h of life.

Montazeri et al. reported distinct BSN level differentiations based on EEG background activity determined from aEEG: BSN of 0–33 (median 14.8, 95% CI 12.8–18.4) corresponds to inactive aEEG, a BSN of 27–61 (41.8, 38.4–43.4) corresponded to burst suppression, 61–91 (75.8, 70.4–73.6) corresponded to continuous normal voltage. The BSN values exceeding 90 were observed in EEG displaying more normal characteristics.^17^ Similar trends were observed based on encephalopathy severity (Fig. 2). We observed that a BSN value of 85 as a potential early biomarker (detectable as early as 6 h after birth) for distinguishing HIE from normal cohort and predicting neurodevelopmental impairment. Additionally, a BSN cutoff of 68 was established to distinguish between non-mild and mild HIE groups. Due to the small number of newborns with HIE before 6 h of life and later than 60 h after birth, the analysis window was restricted to the period between 6 and 60 h after birth. Clinicians could utilize this threshold value as a reference at the bedside to identify newborns at risk. Previous studies utilizing delta power^12^ (DP 147 for C3-C4 AUC = 0.774, 95% CI 0.774, 95% CI 0.631–0.917) and neurovascular coupling^31^ (NVC 10% for C3-C4 AUC = 0.808, 95% CI 0.624–0.991) have reported similar threshold values, which can be valuable biomarkers for clinicians in predicting encephalopathy severity or abnormal outcome on MRI. However, it’s important to note that those studies lacked the inclusion of normal newborns, and their data analysis was conducted offline.

This study has several strengths. First, it evaluates a cloud-based tool that rapidly provides the trend of BSN values at the bedside, enabling immediate clinical decision-making. Second, the tool can identify EEG data with seizures and different types of artifacts that might be hard to identify with a human eye and reject segments with artifacts with visual inspection. Despite the small HIE cohort, the study successfully validated the use of BSN by comparing it with a normal cohort and predicting neurodevelopmental outcomes. It’s worth noting that 67% of our HIE cohort is Hispanic, which highlights the importance of including these underserved and underrepresented patients in research studies. Third, one of the significant advantages of this tool is its easy implementation at the bedside, requiring minimal data processing steps, such as high pass filtering. This simplicity makes it a potential tool to accurately assess the true severity of mild cases and determine the suitability of neuroprotective therapies within the first 6 h of life. Particularly in low-resource settings lacking physician availability for EEG review, BSN could play a crucial role in identifying newborns who would benefit from timely interventions.

Study limitations include the observational nature and small numbers with HIE derived from a single center. However, there is potential to expand the cohort in the future by including collaborating sites in the COOLPRIME trial (https://clinicaltrials.gov/ct2/show/NCT04621279). In this current study, the analysis only considered inter and intra hemispheric central parietal bipolar electrodes. Nevertheless, we and others have shown electrodes in central parietal region to represent global HIE insults.^12,15,23,31–33^ Moreover, Montazeri et al.^17^ developed the BSN algorithm using frontal and parietal electrodes and validated it with frontal and central electrodes, yielding no significant differences in outcomes. This study utilized two different EEG devices (NK and NicoletOne) across cohorts. While bipolar EEG should mitigate the influence of reference electrode and individual system characteristics on aEEG calculation, the potential for systematic variations introduced by these differences may not be ruled out and beyond the scope of this work.

The first TSS was documented within the first 6 h of life, it best coincided with the earliest BSN value of 6–9 h. Given the dynamic nature of HIE injuries, serial assessments were obtained but were not relevant to the need to make decisions in the first day of life regarding treatment selection. Therefore, we restricted our analysis to the earliest BSN values obtained within 6–9 h of life. Ideally, we would have assessed BSN values from birth, but patient care and need to place EEG leads and consent restricted our analysis to this timeframe. The primary goal of the study is to evaluate the association between automatically computed BSN trends during the first 6–9 h of life with neurodevelopmental outcome. The analysis of data beyond this time window is exploratory, aiming to assess whether BSN could be a promising bedside tool for monitoring evolving nature of encephalopathy, especially in newborns with mild HIE to initiate timely interventions.

We recognize cooling device and other equipment generate electrical noise that disrupts the EEG signal, requiring notch filtering. To address these challenges, artifact detection algorithm^27^ was used to identify and remove interference from devices, EMG, movement, electrode pops, and even non-cortical rhythms like ECG. Only clean data segments were used for analysis, ensuring accurate and reliable results. The artifact detection algorithm^27^ effectively identified various artifacts, the use of fixed 50% tolerance threshold presents a limitation but was confirmed with visual checks. This trade-off ensures robust artifact removal while minimizing the data loss, but it might lead to the removal of some valuable data or incomplete removal of certain artifacts. Future research on adaptive threshold could further improve the balance between artifact removal and data retention. While the American Clinical Neurophysiology Society guidelines define seizures lasting 10 s or more as electrographic seizures,^34^ we adopted a more conservative approach in this study. We considered events exceeding 6 s as seizures based on a probability threshold of greater than 0.5, albeit non-empirical. This deviation from the recommended criteria to ensure rigorous seizure removal and maintain the robustness of our analysis.TH can also suppress the EEG background activity, crucial for BSN calculations, making it difficult to differentiate normal variations from those linked to underlying neurological issues.

Due to the relatively small sample size, the variety of equipment, the number of measured time points, and the validation of the model’s performance lacks confirmation from both internal and external datasets. Consequently, the reported findings, including AUC, sensitivity, and specificity, should be interpreted with a degree of caution. The BSN value is calculated using aEEG, and the interpretation might be affected by the specific algorithm used. However, Das et al. did not find any influence of aEEG algorithm in calculation neurovascular coupling.^35^ Finally, neurodevelopmental trajectory needs to be monitored for developing early intervention therapies because true severity of mild HIE may not know until school age.^22,29,36–41^

Future studies will include predicting neurodevelopmental outcome using multi-modal biomarkers include maternal and neonatal characteristics to identify combination of biomarkers that can best distinguish the outcome.

## Conclusion

BSN is a promising bedside tool for monitoring evolving encephalopathy in newborns with HIE and to predict neurodevelopmental outcome. It is comparable to other physiological biomarkers in predicting long-term neurodevelopmental outcome. However, larger studies with predictive modeling are essential, as some measures may be independent predictors of long-term neurodevelopmental outcomes. Adding BSN to a multimodal bedside monitor may assist clinicians in making prompt treatment decisions to reduce long-term neurodevelopmental impairment. This could help identify those who may benefit from additional interventions and protect those who may not require TH, thereby preventing potential adverse effects on their neurodevelopmental outcome.

## Supplementary Information

Supplementary information

## Data Availability

Request for access to the data should be directed to the corresponding author with a reasonable request, and the data will be shared in accordance with applicable data protection and confidentiality guidelines.
