# Supplementary material for: Prognostic value of quantitative EEG in early hours of life for neonatal encephalopathy and neurodevelopmental outcomes
Source: Pediatr Res. Author manuscript; Available in PMC 2024 Oct 25. (PMC11499260; doi:10.1038/s41390-024-03255-8)
Supplement: supplementary material [file NIHMS2011528-supplement-supplementary_material.pdf]

Supplementary Information

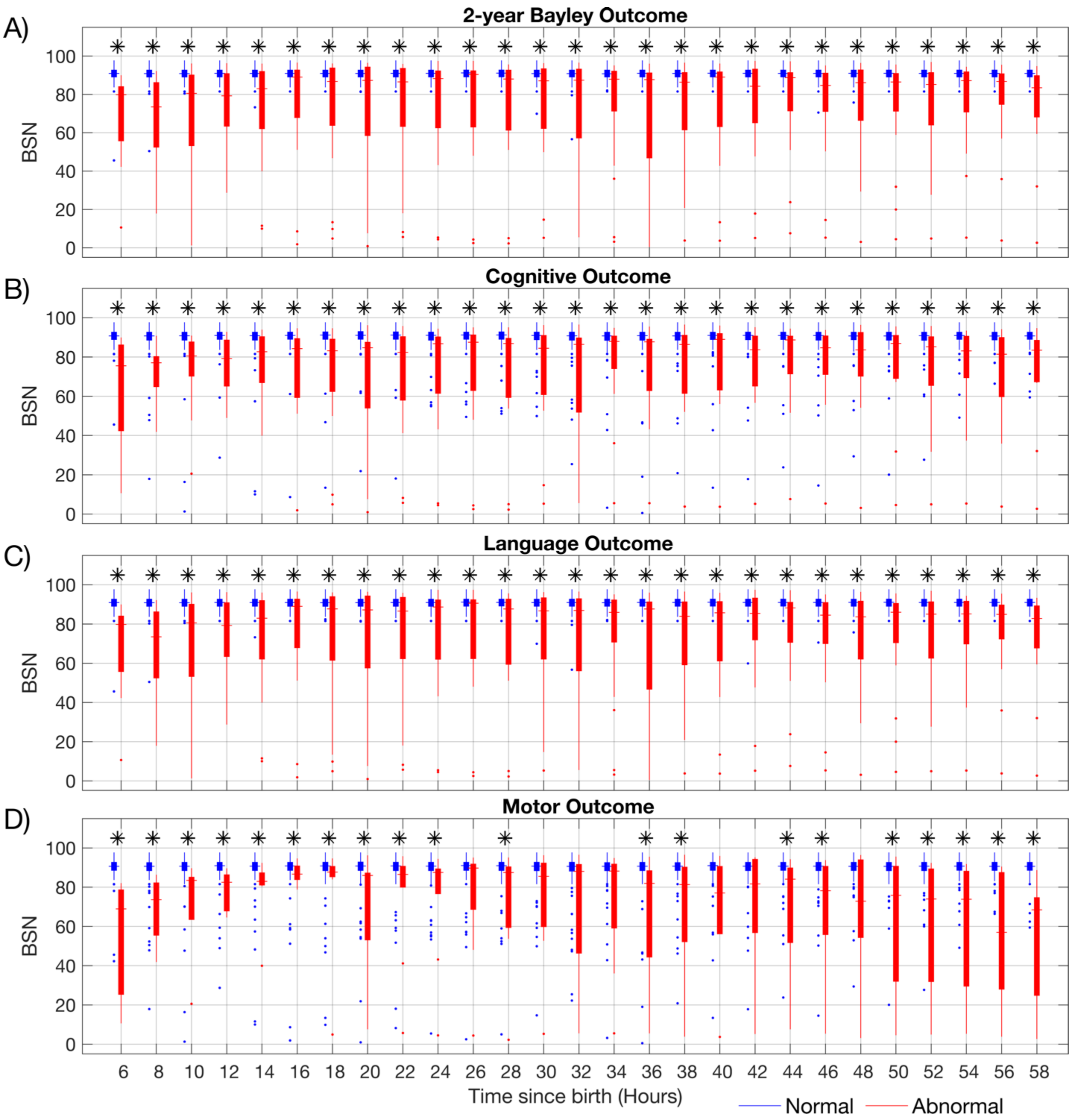

Figure S1. Distribution of mean BSN values over 2-hour windows from 6 to 60 hours hour since birth for normal and abnormal 2-year Bayley (A), cognitive (B), language (C), and motor (D) outcomes. Abnormal outcome was defined as death or 2-year Bayley scores < 85 in any of cognitive, language, or motor domains. An asterisk on the top of each figure indicates that the BSN values for infants with normal outcome are significantly greater than those for infants with abnormal outcomes, as determined by the Wilcoxon Rank-sum test.

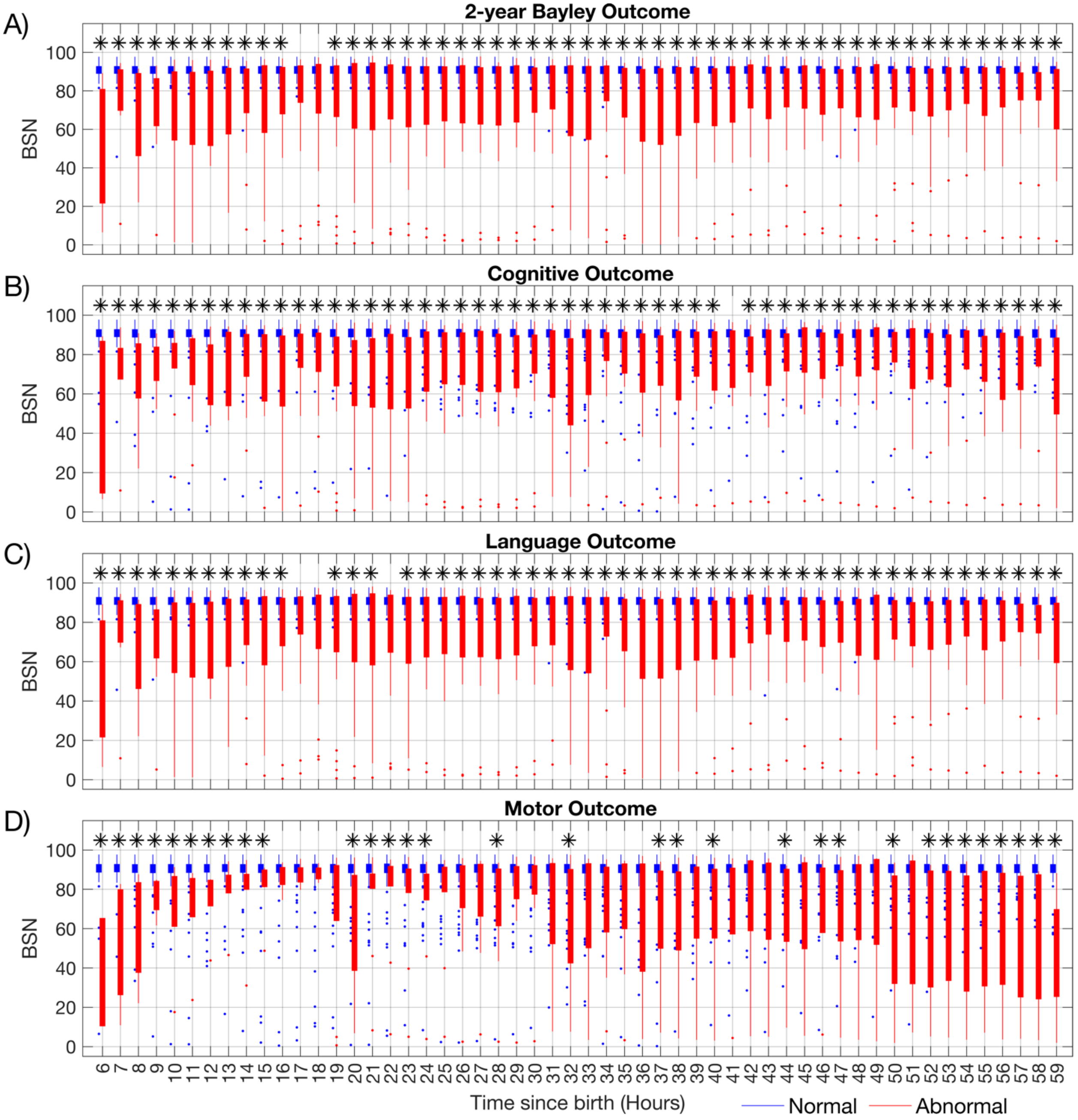

Figure S2. Distribution of mean BSN values over 1-hour windows from 6 to 60 hours since birth for normal and abnormal 2-year Bayley (A), cognitive (B), language (C), and motor (D) outcomes. Abnormal outcome was defined as death or 2-year Bayley scores < 85 in any of cognitive, language, or motor domains. An asterisk on the top of each figure indicates that the BSN values for infants with normal outcome are significantly greater than those for infants with abnormal outcomes, as determined by the Wilcoxon Rank-sum test.

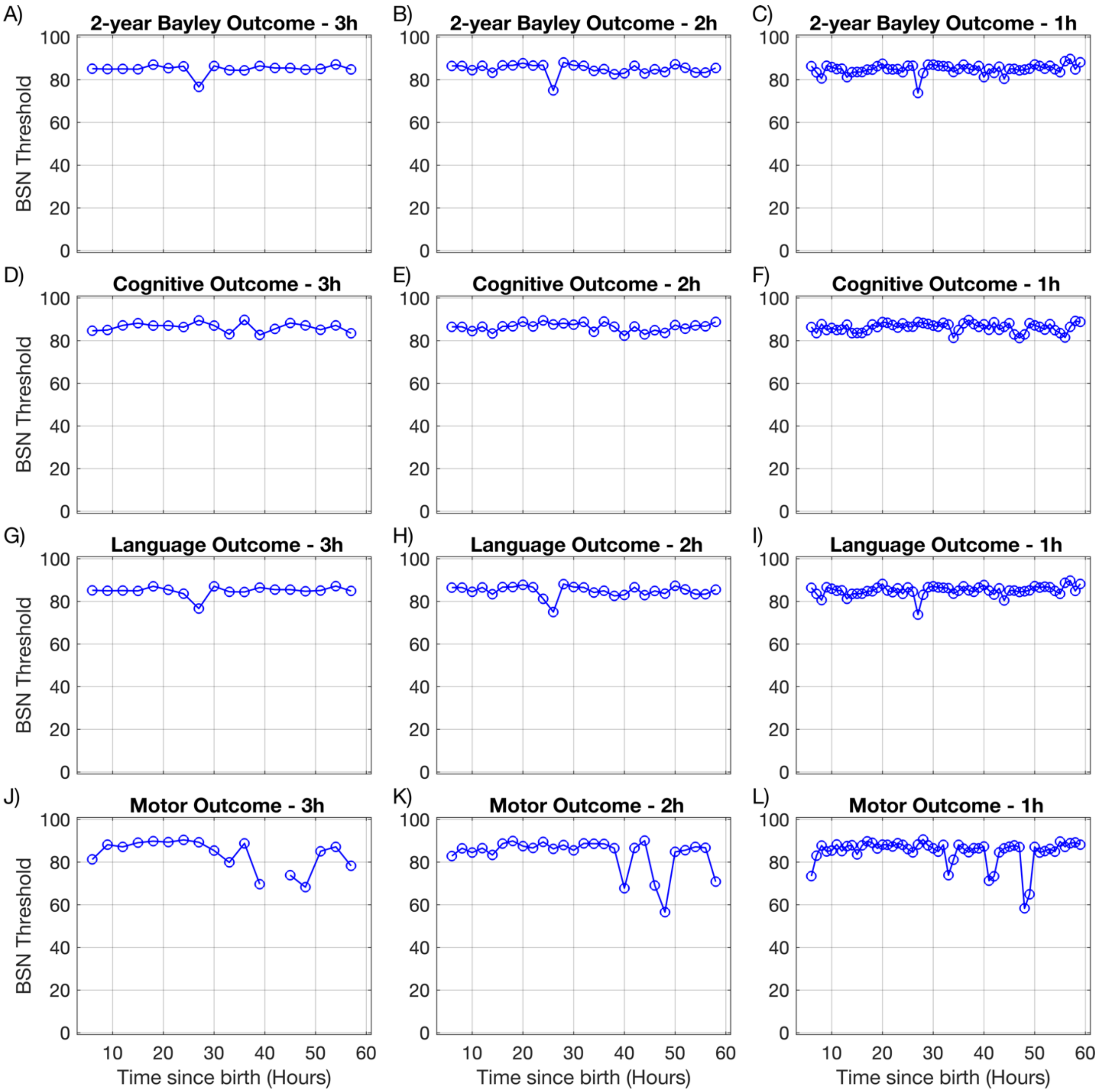

Figure S3. To evaluate the impact of effect of time window duration on analysis, BSN values were averaged over 3-, 2-, and 1-hour windows. Receiver operating characteristic (ROC) analysis identified a BSN threshold of 80 that distinguished normal from abnormal outcomes for 2-year Bayley, cognitive, language, and motor outcomes from 6 to 60 hours since birth, except for motor outcomes between 39 and 52 hours since birth. There was little or no difference in BSN thresholds between averaging BSN over three different time windows, suggesting that BSN data can be analyzed in short intervals.

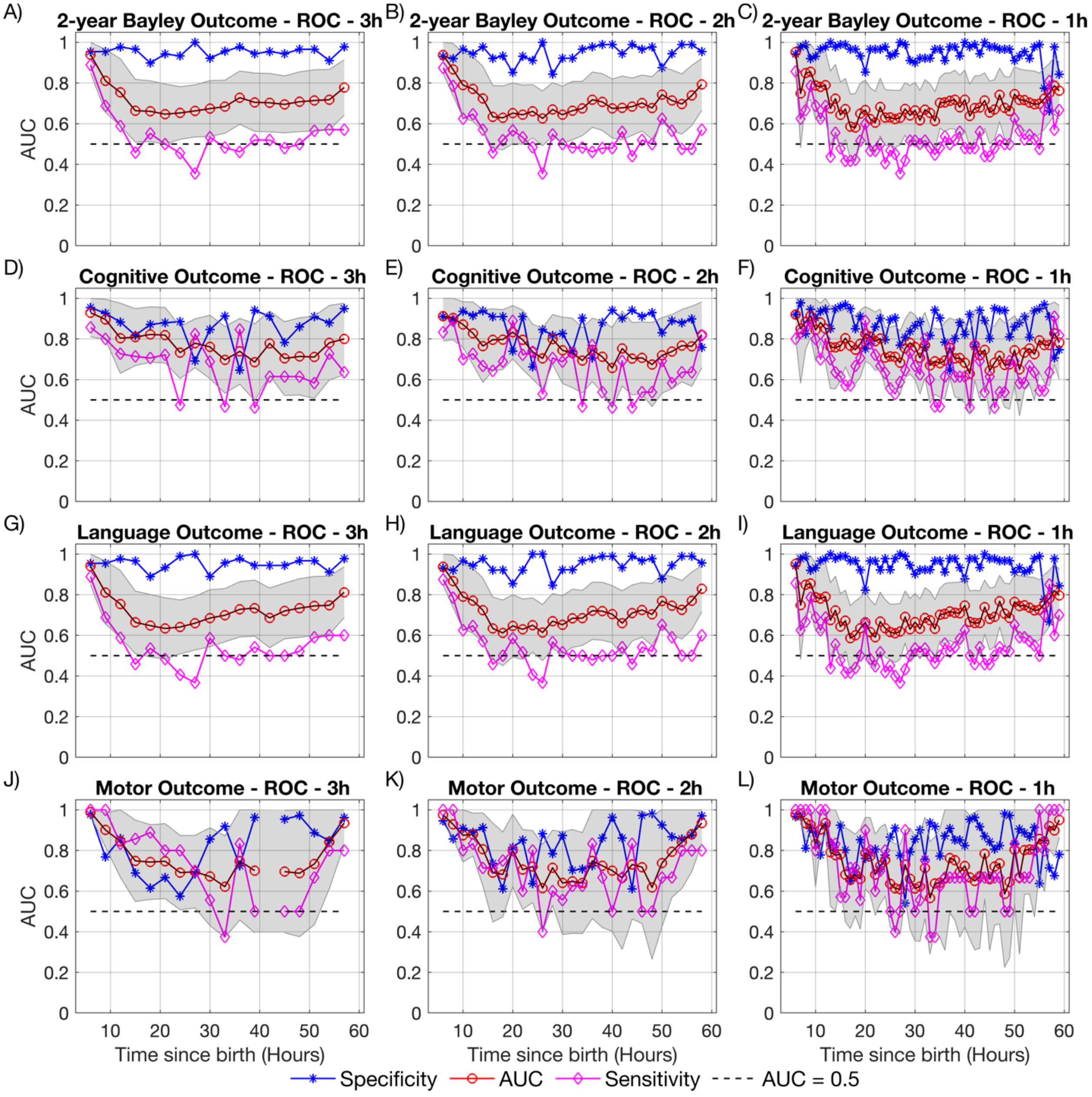

Figure S4. To evaluate the effect of time window duration on analysis, BSN values were averaged over 3-, 2-, and 1-hour windows. Receiver operating characteristic (ROC) analysis showed that the area under the curve (AUC) was greater than 0.5 for 2-year Bayley, cognitive, language, and motor outcomes from 6 to 60 hours since birth except for motor outcome between 49 and 52 hours since birth for 3-hour time window. There was little to no difference in AUC values between averaging BSN over three different time windows, suggesting that BSN data can be analyzed in short intervals.

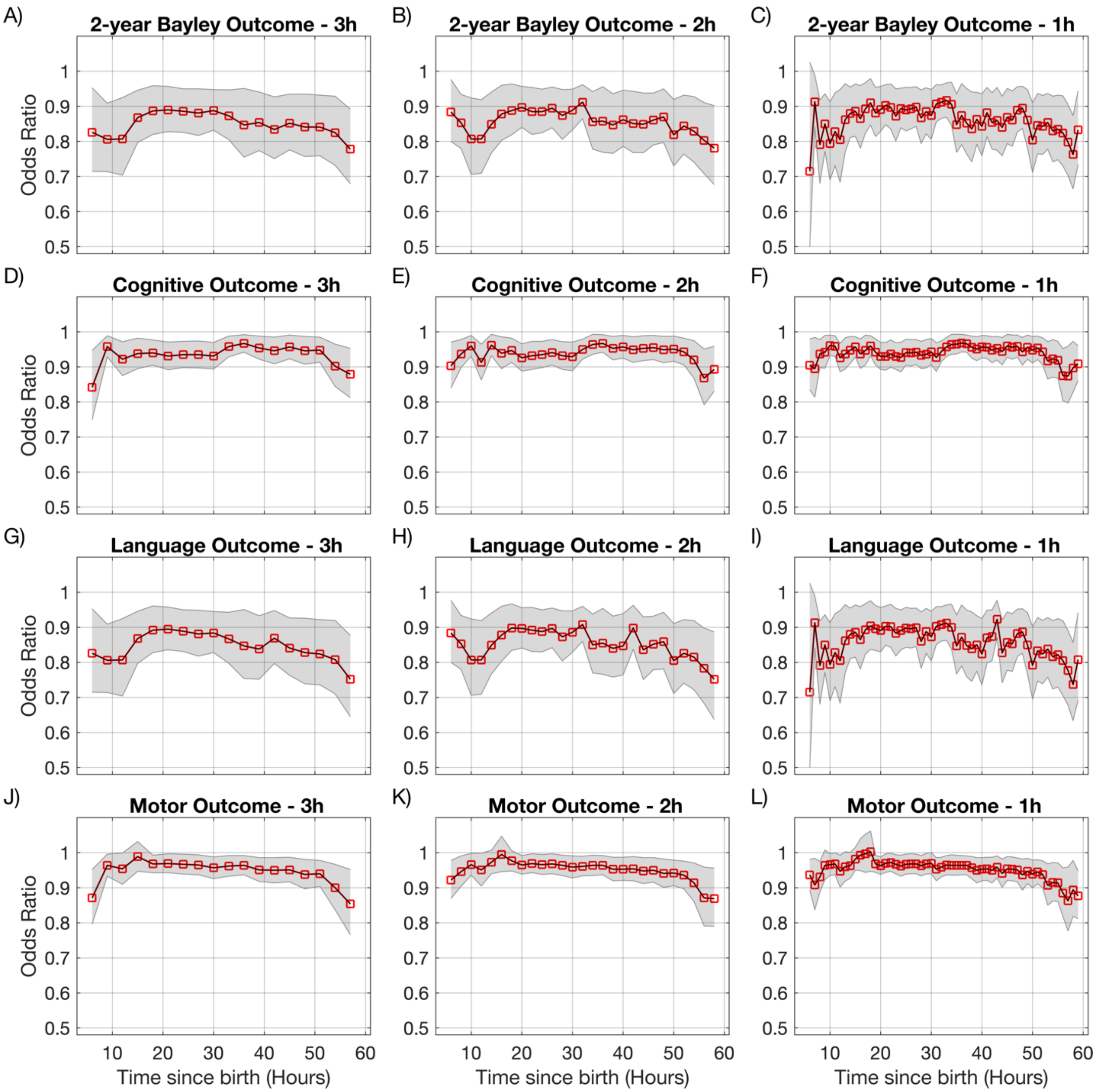

Figure S5. Univariate logistic regression analysis of BSN values averaged over 3-, 2-, and 1-hour windows revealed little to no difference in odds ratios (ORs) for 2-year Bayley, cognitive, language, and motor outcomes at different time points after birth (6-60 hours). Shaded areas indicate 95% confidence intervals (CIs) for ORs. Higher BSN values were significantly associated with lower odds of abnormal outcomes. These findings suggest that BSN data can be analyzed in short intervals.

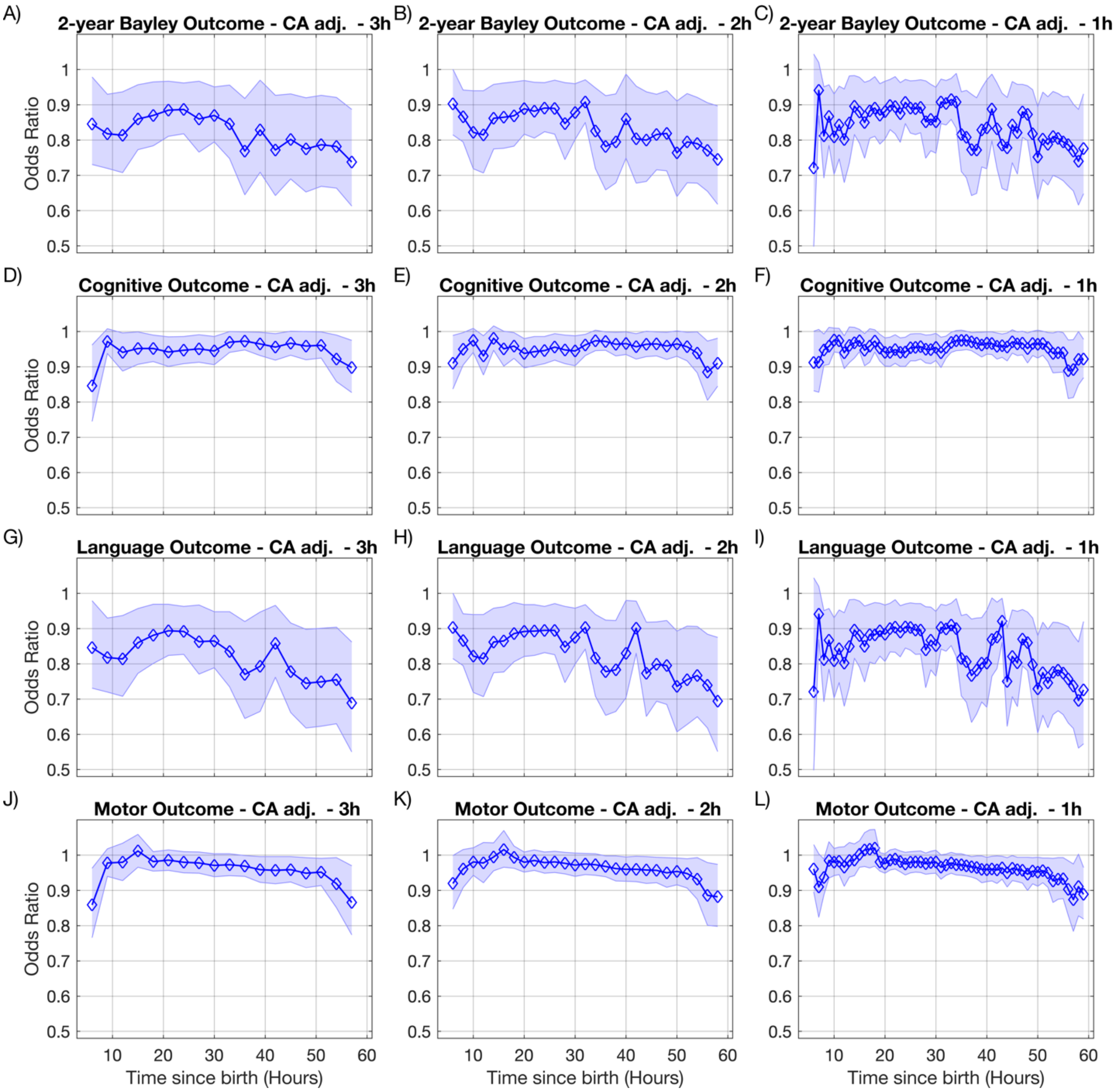

Figure S6. Conceptual ages of the control and HIE cohorts were 40 [39, 42] and 39 [38, 40], respectively. Univariate logistic regression analysis was adjusted for conceptual age to test the age effect. BSN values averaged over 3-, 2-, and 1-hour windows revealed little to no difference in odds ratios (ORs) for 2-year Bayley, cognitive, language, and motor outcomes at different time points after birth (6-60 hours). Shaded areas indicate 95% confidence intervals (CIs) for ORs. Higher BSN values were significantly associated with lower odds of abnormal outcomes. These findings suggest that BSN data can be analyzed in short intervals.

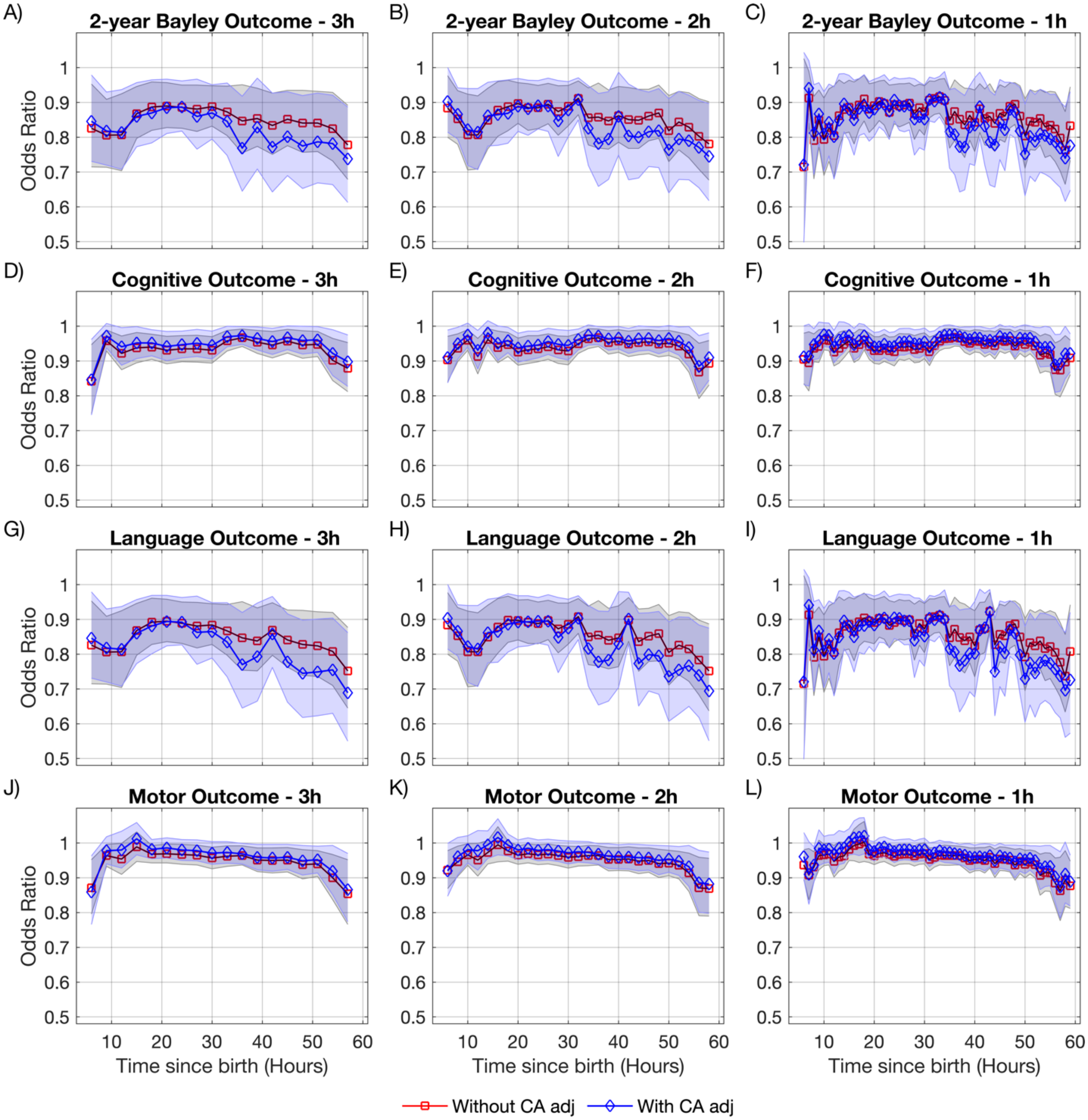

Figure S7. Conceptual ages of the control and HIE cohorts were 40 [39, 42] and 39 [38, 40], respectively. Univariate logistic regression analysis was performed without and with adjustment for conceptual age. Little to no difference was observed in odds ratios (ORs) for 2-year Bayley, cognitive, language, and motor outcomes at different time points from 6 to 60 hours after birth, after adjusting for conceptual age.
